# Acute Kidney Injury in Heart Failure Revisited—The Ameliorating Impact of “Decongestive Diuresis” on Renal Dysfunction in Type 1 Acute Cardiorenal Syndrome: Accelerated Rising Pro B Naturetic Peptide Is a Predictor of Good Renal Prognosis

**DOI:** 10.3390/jcm6090082

**Published:** 2017-08-29

**Authors:** Macaulay Amechi Chukwukadibia Onuigbo, Nneoma Agbasi, Mohan Sengodan, Karen Flores Rosario

**Affiliations:** 1 College of Medicine, Mayo Clinic, Rochester, MN 55905, USA; 2 Department of Nephrology, Mayo Clinic Health System, Eau Claire, WI 54702, USA; 3 North East London NHS Foundation Trust, Ilford, Essex 1G3 8XJ, UK; nneomaa@hotmail.com; 4 Hospital Medicine, Mayo Clinic Health System, Eau Claire, WI 54702, USA; sengodan.mohan@mayo.edu (M.S.); kpflores@wisc.edu (K.F.R.); 5 Medical School, University of Wisconsin School of Medicine and Public Health, Madison, WI 53726, USA

**Keywords:** acute decompensated heart failure, cardiorenal syndrome, central venous pressure, congestive heart failure, congestive renal failure, continuous intravenous furosemide infusion, fluid balance, intravenous chlorothiazide, intravenous decongestive diuresis, Pro B Naturetic Peptide, renal venous pressure, renal venous hypertension, serum creatinine, ultrafiltration

## Abstract

There is mounting evidence that forward heart failure as manifested by low cardiac output alone does not define the degree of renal dysfunction in cardiorenal syndrome. As a result, the term “congestive renal failure” was coined in 2012 by Ross to depict the role of renal venous hypertension in type 1 acute cardiorenal syndrome. If so, aggressive decongestive therapies, either through mechanical ultrafiltration with dialysis machines or pharmacologic ultrafiltration with potent diuretics, would lead to improved cardio and renal outcomes. Nevertheless, as recently as 2012, a review of this literature had concluded that a renal venous hypertension-directed approach using diuretics to manage cardio-renal syndrome was yet to be fully investigated. We, in this review, with three consecutive case series, describe our experience with pharmacologic decongestive diuresis in this paradigm of care and argue for studies of such therapeutic interventions in the management of cardiorenal syndrome. Finally, based on our observations in the Renal Unit, Mayo Clinic Health System, in Northwestern Wisconsin, we have hypothesized that patients with cardiorenal syndrome presenting with accelerated rising Pro B Naturetic Peptide levels appear to represent a group that would have good cardio- and renal-outcomes with such decongestive pharmacologic therapies.

## 1. Introduction

### Heart Failure, Renal Failure and the Role of Renal Venous Hypertension in Cardiorenal Syndrome: A Review of the Current Literature

There is mounting evidence that forward heart failure as manifested by low cardiac output alone does not define the degree of renal dysfunction in cardiorenal syndrome [1,2,3,4,5,6,7]. Consequently, Ross in 2012 referred to kidney dysfunction associated with heart failure as “congestive renal failure” [1]. In an analysis of the ADHERE (Acute Decompensated Heart Failure National Registry) database of 118,465 decompensated heart failure admissions, Heywood et al. could not demonstrate an association between left ventricular systolic dysfunction and renal impairment [2]. Moreover, in the ESCAPE (Evaluation Study of Congestive Heart Failure and Pulmonary Artery Catheterization Effectiveness) trial involving hospitalized decompensated heart failure patients, kidney function did not correlate with cardiac index, pulmonary capillary wedge pressure, or systemic vascular resistance, but rather was (weakly) associated with right atrial pressure [3]. Furthermore, Damman et al.’s retrospective analysis of 2557 patients undergoing right heart catheterization demonstrated that elevated central venous pressure (CVP) was associated with low estimated glomerular filtration rate (eGFR) independently from cardiac index, and it predicted mortality [4]. Another set of investigators reported supportive findings in a study of 2647 patients with systolic heart failure, in which depressed eGFR and mortality were associated with congestive findings such as ascites and elevated jugular venous pressure [5]. Likewise, Guglin et al. described catheterization findings in 178 HF patients wherein low eGFR correlated with high CVP and low renal perfusion pressure (mean arterial pressure minus CVP), but not the cardiac index or left ventricular ejection fraction (LVEF) [6]. Similarly, in Maeder et al.’s report of 196 heart failure patients, the echocardiographic severity of tricuspid regurgitation was independently associated with the degree of renal dysfunction [7]. The conclusion was that although a causal relationship cannot be proven, significant tricuspid regurgitation contributes to renal dysfunction in heart failure patients, probably by elevation of central and renal venous pressure [7].

From the foregoing therefore, although experimental, animal and human studies have for more than half a century supported the concept that backward failure with increased renal venous hypertension is a potent etiopathogenetic factor in the causation of renal failure associated with decompensating heart failure, as recently as 2012, a review of this literature had concluded that a renal venous hypertension-directed approach using diuretics to manage cardio-renal syndrome was yet to be fully investigated [1]. This review called for larger and more rigorous trials to definitely establish under what circumstances conventional pharmacologic and ultrafiltration goals might best be directed towards central venous pressures rather than left ventricular or cardiac output parameters [1].

The mission of this manuscript is to describe our experience with three patients presenting to the Renal Unit, Mayo Clinic Health System, Eau Claire, in Northwestern Wisconsin, over three months, May–June 2017, with features of worsening symptomatic congestive heart failure or acute decompensating heart failure with concurrent worsening acute kidney injury (cardiorenal syndrome) and the response of these patients to conventional pharmacologic diuresis, leading to improvement in both heart failure symptoms and acute kidney injury. Following that is a discussion of the utility and pharmacodynamics of combination intravenous Thiazide-Loop diuretics in this paradigm of care. Such therapeutic maneuvers would prove invaluable to practitioners in resource-poor settings without access to mechanical ultrafiltration with dialysis or similar equipment.

## 2. Case Reports

### 2.1. Case 1

In early March 2017, the Nephrology service was consulted to see an overweight 83-year-old female patient admitted to the Hospitalist service a week earlier with dyspnea and volume overload from symptomatic heart failure complicated by rapid ventricular response atrial fibrillation, against a background history of hypertension and stage 3 CKD. Other pertinent past medical history include hypertension, sarcoidosis, COPD, atrial fibrillation and type 2 diabetes mellitus. A month earlier, she was discharged after 5 days with symptomatic hypotension and dizziness associated acute kidney injury with peak creatinine of 2.21 mg/dL, and then associated with UTI that was treated with Bactrim. At the time she was also on Lisinopril, which was then discontinued. Subsequently following conservative management in the hospital, kidney function as measured by the serum creatinine had returned to near baseline of 0.96 mg/dL at discharge.

This time in March 2017, she had become increasingly short of breath and hypotensive as well and serum creatinine had risen back up again to as high as 1.79 mg/dL at the time of the Nephrology consultation. Temperature was 36.9 °C, heart rate 67 per/min, respiratory rate 18 per/min, oxygen saturation 90% on room air, and blood pressure was 106/60. She weighed up to 75.4 kg, height was 163 cm, with a BMI of 28.4 kg/m^2^. She was dyspneic, JVD was elevated, with 2+ bilateral extremity edema and reduced breath sounds in both lung bases bilaterally, with bibasilar inspiratory rales evident posteriorly. Admission chest radiograph showed cardiomegaly, new bilateral pleural effusions, pulmonary vascular congestion and hypoventilation. Echocardiogram revealed normal left ventricular systolic function with ejection fraction of 64%, mild mitral valve regurgitation, bi-atrial enlargement, normal right ventricular function with right ventricular systolic pressure of 29 mmHg. At the time of this consultation, serum albumin was 2.8 g/dL, sodium was 134 mmol/L, potassium 3.6 mmol/L, chloride 94 mmol/L, bicarbonate 24 mmol/L, calcium 8.9 g/dL, hematocrit 24.9%, WBC, platelets, TSH, Free T4, AST, ALT and total bilirubin were all normal. Pro Pro B Naturetic Peptide level that was elevated at 581 pg/mL on 2 February 2017 had rapidly escalated to 4907 pg/mL by 23 February 2017, three weeks later, just prior to this admission (Pro Pro B Naturetic Peptide reference range ≤ 450 pg/mL).

Earlier in the admission, she was started on intravenous amiodarone for rate control of atrial fibrillation. Furthermore, oral Diltiazem, 120 mg three times a day, was subsequently discontinued when she became hypotensive. She had been started on continuous intravenous Furosemide infusion at 10 mg/h but the previous 24-h urine output was only 1500 cc.

**Figure 1:**
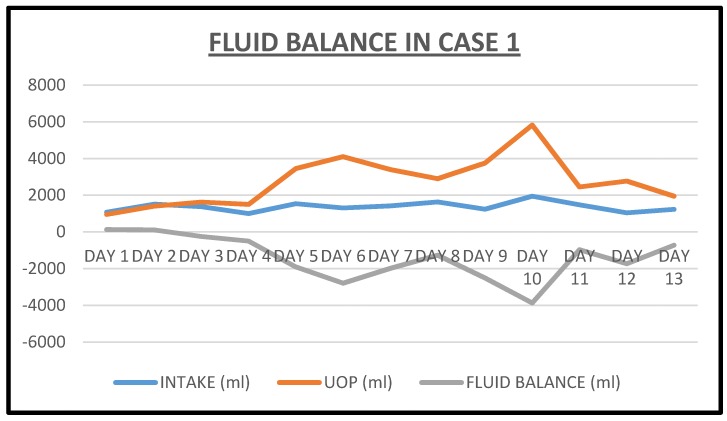
Fluid balance with total intake and urine output in Case 1 during the index admission.

**Figure 2:**
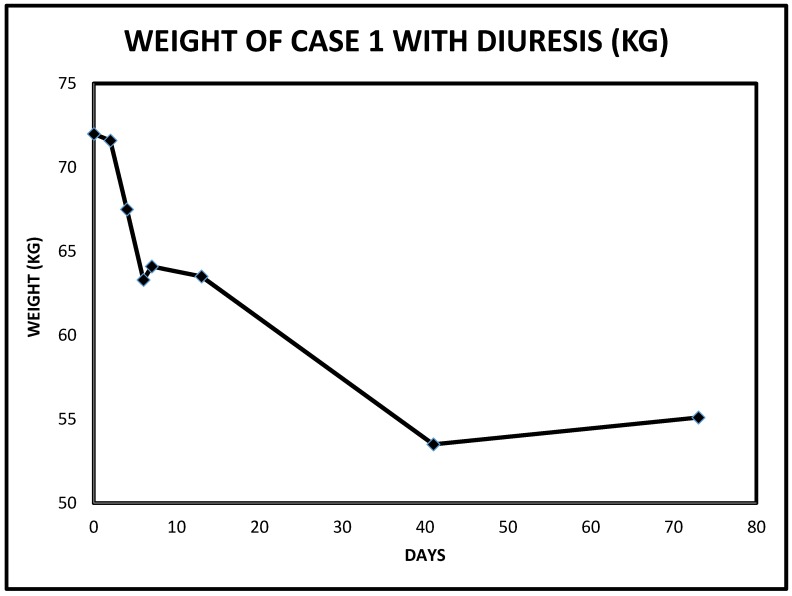
Weight changes following intravenous decongestive diuresis in Case 1.

**Figure 3:**
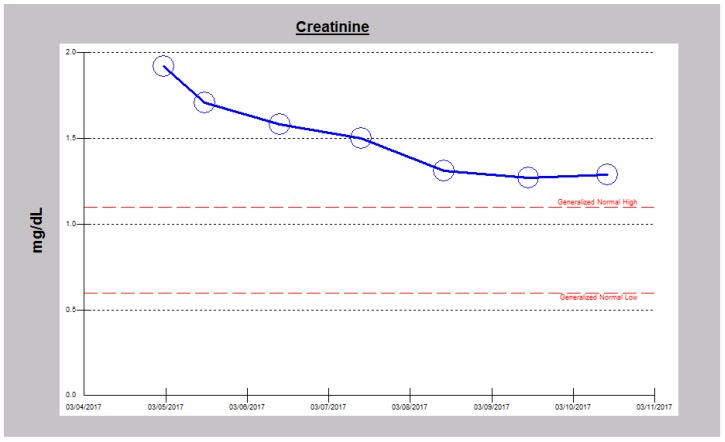
Falling serum creatinine in Case 1 following intravenous decongestive diuresis, 4–11 March 2017.

**Figure 4:**
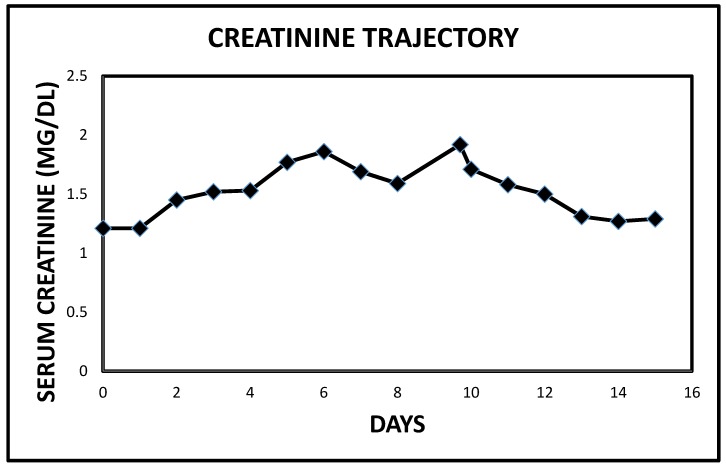
Serum creatinine trajectory in Case 1 leading to and following the use of intravenous decongestive diuresis, 4–11 March 2017.

We doubled the dose of intravenous continuous infusion of Furosemide to 20 mg/h and added intravenous Chlorothiazide 500 mg every 12 h. The chlorothiazide was discontinued after one dose when the patient raised concerns for a history of drug-induced pancreatitis. We then increased the dose of intravenous Furosemide to 25 mg/h, added daily intravenous Albumin 25 gm infusion to run concurrently with the continuous Furosemide infusion because of severe hypoalbuminemia. The edematous legs and feet were elevated and wrapped with ACE wraps. Overnight, urine output had more than doubled to 3450 cc (Figure 1). Subsequently, twenty-four urine output increased to over 3–4 L (Figure 1). After 5 days of the Furosemide infusion, she was switched to oral Bumetanide 2 mg twice daily. She continued to make urine in excess of 2.5 L an day and was discharged after another two days, much improved with edema, dyspnea and orthopnea resolved, together with significant weight loss (Figure 2). She had achieved a weight loss of 12 kg during the last nine days of the admission. Her serum creatinine trajectory also improved during the second half of this admission and serum creatinine at discharge was 1.29 mg/dL (Figure 3 and Figure 4).

### 2.2. Case 2

**Figure 5:**
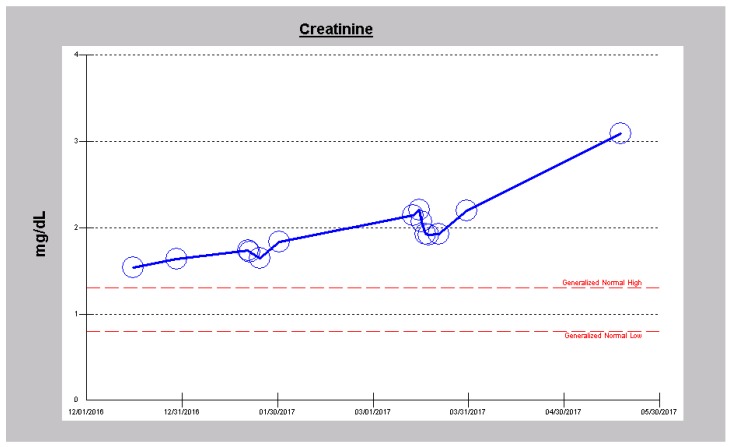
Worsening serum creatinine trajectory in Case 2 leading to the admission in mid-May 2017.

In mid-May 2017, an 84-year old Caucasian male was readmitted to our hospital, after two months, from a Nursing Home with worsening shortness of breath, orthopnea, and inability to get around his assisted living facility despite the use of 24/7 nasal cannula oxygen at 2 L/min. Past medical history was significant for severe oxygen-dependent COPD in an ex-smoker, chronic diastolic heart failure, obstructive sleep apnea, type II diabetes mellitus, atrial fibrillation, hypertension, tricuspid regurgitation, cor pulmonale with worsening pulmonary hypertension, previous coronary artery bypass and chronic kidney disease stage IIIB with serum creatinine of 1.93 mg/dL (eGFR = 33 mL/min/1.73 sq. m BSA in late March 2017). Outpatient medications included Metoprolol 25 mg twice daily, insulin and Torsemide 10 mg twice daily. In the Emergency Department, his oxygen saturation was 87% on 2 L per minute nasal cannula oxygen. Temperature was 36.4 °C, pulse was 76/min, respiratory rate 22/min and BP was 120/87. His oxygen saturation improved to 97% on high flow oxygen. He weighed 75 kg, height was 193 cm, with a BMI of 20.1 kg/m^2^. Jugular venous distension was present and he had bilateral pitting pretibial edema. The lungs revealed bilateral basal inspiratory crepitations. Hemoglobin was 13.3 g/dL, Pro B Naturetic Peptide level was grossly elevated at 38,547 pg/mL (<450), from a level of 16,711 pg/mL in January 2017 (Pro B Naturetic Peptide reference range ≤ 450 pg/mL). Simultaneously, the serum creatinine had increased from a baseline of 1.65 mg/dL in January 2017 to 3.09 mg/dL (Figure 5). Other pertinent laboratory indices were chloride 91 mmol/L, bicarbonate 31 mmol/L but with otherwise normal other electrolytes. Chest radiograph revealed cardiomegaly, hypoventilation, bibasilar atelectasis and pulmonary vascular congestion. On echocardiogram, the left ventricular function was preserved at 55–60% but the right ventricular systolic pressure had risen from 64 mm Hg in December 2016 to 72 mm Hg in mid-May 2017, together with severe right atrial enlargement.

**Figure 6:**
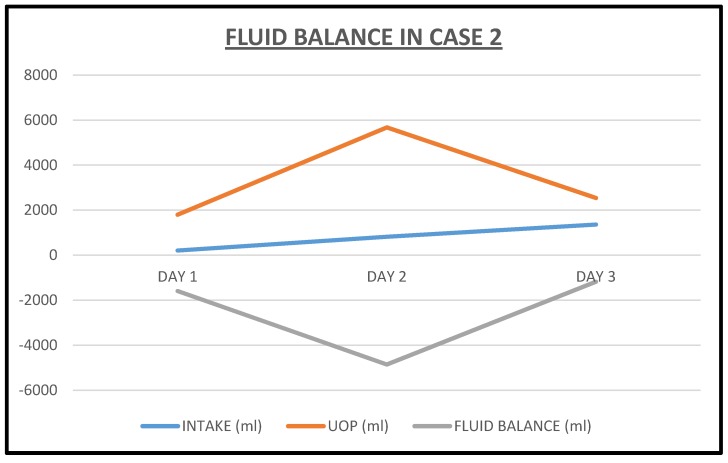
Fluid balance with total intake and urine output in Case 2 during the index admission.

**Figure 7:**
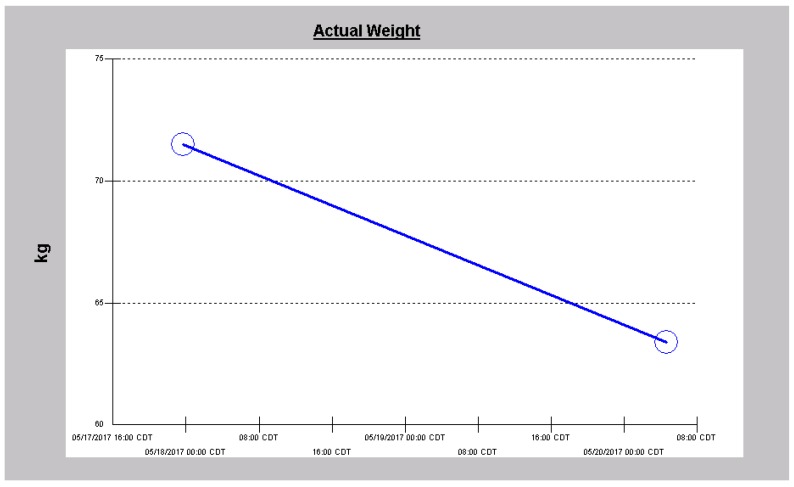
Weight reduction following intravenous decongestive diuresis in Case 2.

**Figure 8:**
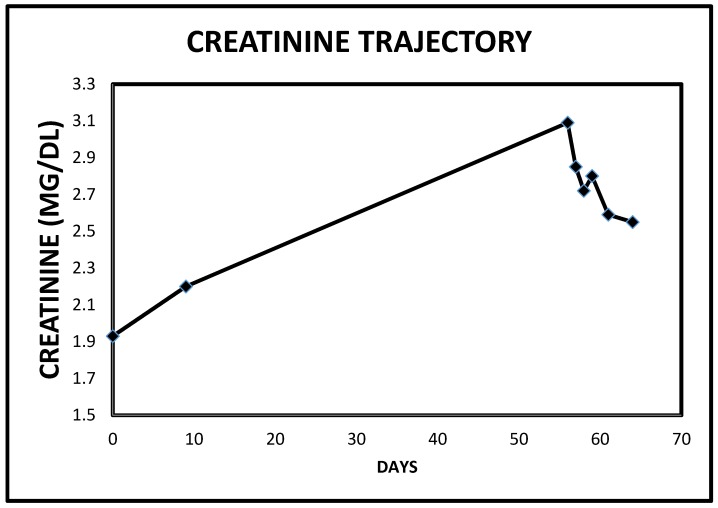
Serum creatinine trajectory in Case 2 following the use of intravenous decongestive diuresis.

He was started on continuous infusion Furosemide at 20 mg/h, together with concurrent intravenous Chlorothiazide 500 mg every 8 h. Overnight, in just over 12 h, he had made 1.8 L of urine with only 207 cc as intake. Over the next two days, diuresis continued with increasing urine output (Figure 6) and decreasing weight (Figure 7). He continued to improve and by the third admission day, both intravenous diuretics were discontinued. Leg edema had resolved, dyspnea had stabilized and serum creatinine had continued to improve (Figure 8). 

He was switched to oral Torsemide, 20 mg twice daily, together with oral Metolazone, 2.5 mg every other day, as an adjunct diuretic agent. Foley catheter was removed and he was discharged much improved to the Nursing Home. Nevertheless, two days after his discharge to the Nursing Home, he became increasingly weak, despite improved dyspnea and still improving kidney function. The patient at this point, with the support of the family, in view of all the concurrent comorbidities, opted for hospice care and he peacefully passed away about a week afterwards.

### 2.3. Case 3

A morbidly obese 62-year old Caucasian male patient was transferred from a Nursing Home in June 2017 by ambulance with worsening dyspnea, oxygen desaturation of about 70% and hypotension to our Emergency Department. Past medical history was significant for morbid obesity, likely sleep apnea, chronic hypercapnic respiratory failure from COPD, peripheral vascular disease, venous insufficiency, generalized debility, preserved left ventricular ejection fraction of 72%, and hypertension with early stage II CKD, with baseline serum creatinine of less than 1 mg/dL. Pertinent outpatient medications included potassium chloride 40 mEq twice daily, spironolactone 25 mg daily which was started two days before this admission, Furosemide 20 mg twice daily, Duonebs, baby Aspirin, Gabapentin 600 mg twice daily, Oxycodone 10 mg extended release twice daily, pantoprazole and Atorvastatin. The patient had undergone an aortogram with run off followed by a left lower extremity angioplasty for a high-grade proximal left femoral-popliteal bypass in situ vein stenosis two days prior to the admission—he had required temporary intubation for this procedure that was otherwise successful and without other complications.

**Figure 9:**
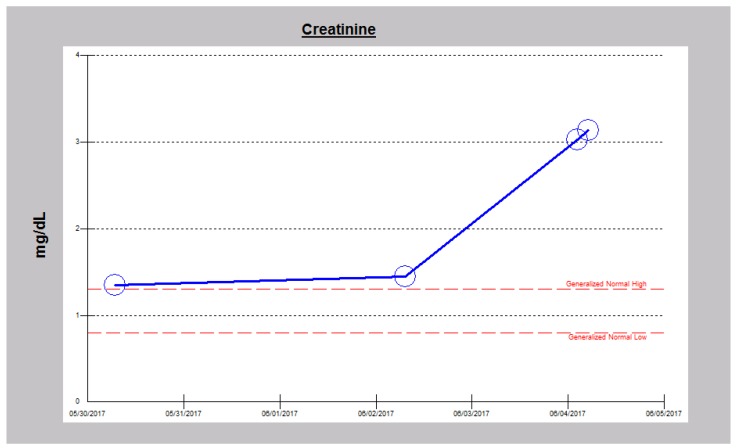
Worsening serum creatinine trajectory in Case 3 leading to the admission in early June 2017.

Initial blood pressure was 88/63, pulse 71/min and oxygen saturation quickly improved to 99% on BIPAP. He weighed 133.5 kg, height was 190 cm, with a BMI of 36.84 kg/m^2^. He had 2+ bilateral lower extremity edema, and chest examination revealed reduced breath sounds with bibasilar inspiratory rales heard posteriorly. Serum creatinine was 3.14 mg/dL, a significant increase from a week previously (Figure 9). Other pertinent laboratory indices were serum potassium 7 mmol/L, serum bicarbonate 27 mmol/L, phosphorus 7.1 mg/dL with a normal anion gap of 13 mmol/L. He was in respiratory acidosis and respiratory failure with arterial blood gas pH of 7.18, pCO_2_ of 84 mm Hg, and bicarbonate of 30 mmol/L. His Pro B Naturetic Peptide level that was nearly normal in August 2016 at 151 pg/mL was grossly elevated at 1426 pg/mL in mid May 2017, and had further escalated to 5503 pg/mL on the day of admission in June 2017 (Pro B Naturetic Peptide reference range ≤ 125 pg/mL). There was in addition, mild rhabdomyolysis with total CK of 1720 U/L, ALT was normal at 17 U/L but AST was mildly elevated at 54 U/L. Lactic acid was normal. EKG was abnormal but the first degree AV block, possible inferior infarct and prolonged QT interval were not new and T waves were not prominent. Chest radiograph revealed worsening cardiomegaly when compared to October 2016, hypoventilation, pulmonary vascular congestion and basal atelectatic changes without overt infiltrates evident. Echocardiogram was not available. He received two 500 cc boluses of normal saline and his blood pressure stabilized with SBP > 120 mm Hg. Other emergent therapies for the hyperkalemia included Ipratropium-Albuterol nebulizer treatments, two ampoules of intravenous Dextrose 50 (25 gm each) infusions, intravenous regular insulin and oral Kayexalate, 15 gm in 60 mL given once [8].

Nephrology service was consulted. Evaluation confirmed gross 2+ to 3+ bilateral lower extremity edema, ascites, anasarca with reduced breath sounds with bibasilar inspiratory rales heard posteriorly. A Foley catheter was inserted due to evidence of urinary retention. The working diagnosis was contrast-induced nephropathy, further complicated by severe dyspnea likely the combination of chronic obstructive pulmonary exacerbation, narcotic/Gabapentin toxicity, with volume overload and diastolic congestive heart failure, given an otherwise normal echocardiogram in August 2016 with left ventricular ejection fraction of 72%. Spironolactone, Oxycodone, Gabapentin and potassium chloride were promptly discontinued. Continuous intravenous Furosemide infusion was started at 20 mg/h together with concurrently administered intravenous Chlorothiazide 500 mg every 8 h, and serum creatinine and electrolytes were monitored every 6 h. In addition, oral Doxycycline for longstanding cough and discolored sputum production, together with oral Prednisone and inhaled steroids were added later during the admission. He also went back on the BiPaP machine, which had been discontinued by the patient some months earlier for unclear reasons.

**Figure 10:**
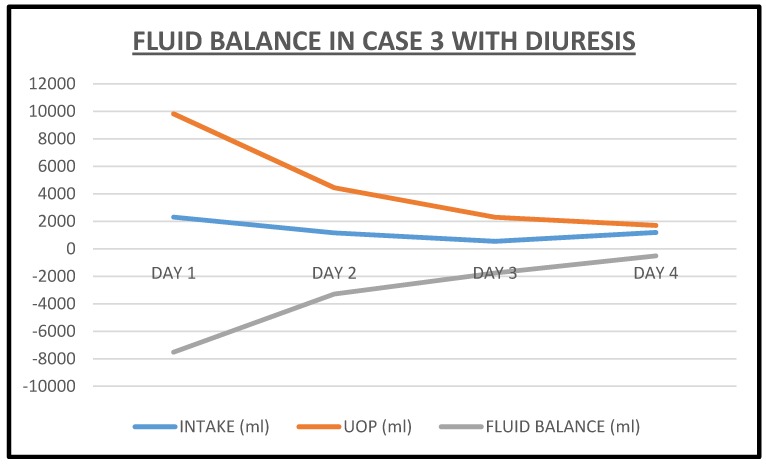
Fluid balance with total intake and urine output in Case 3 during the index admission.

**Figure 11:**
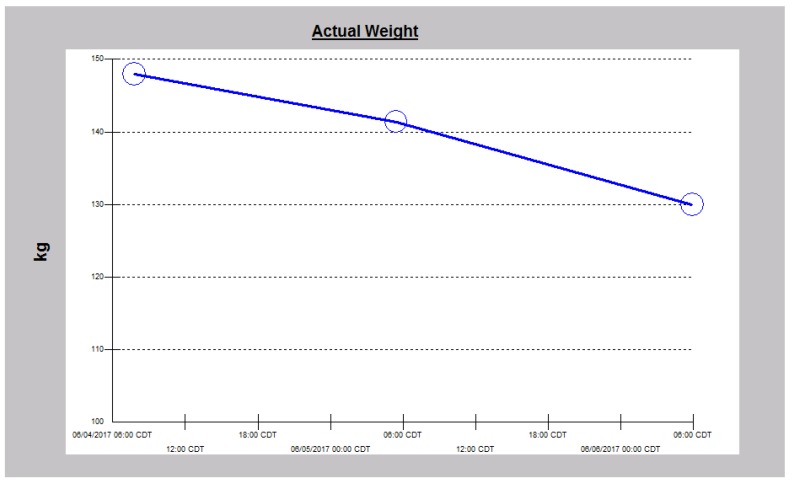
Weight reduction following intravenous decongestive diuresis in Case 3.

**Figure 12:**
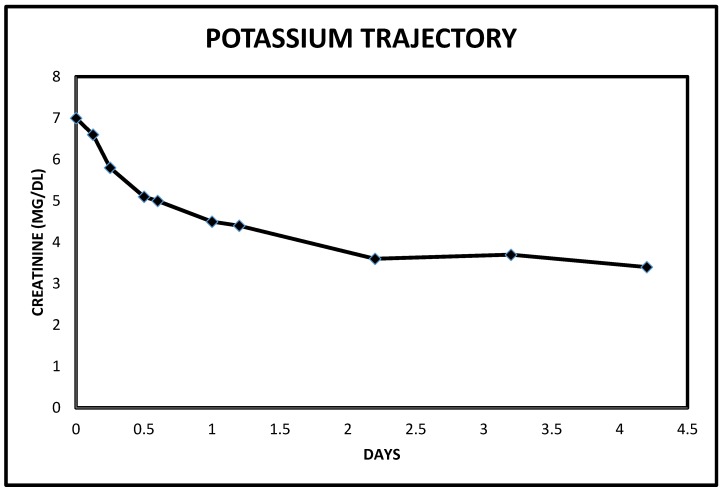
Normalized serum potassium from hyperkalemic levels following intravenous decongestive diuresis in Case 3.

**Figure 13:**
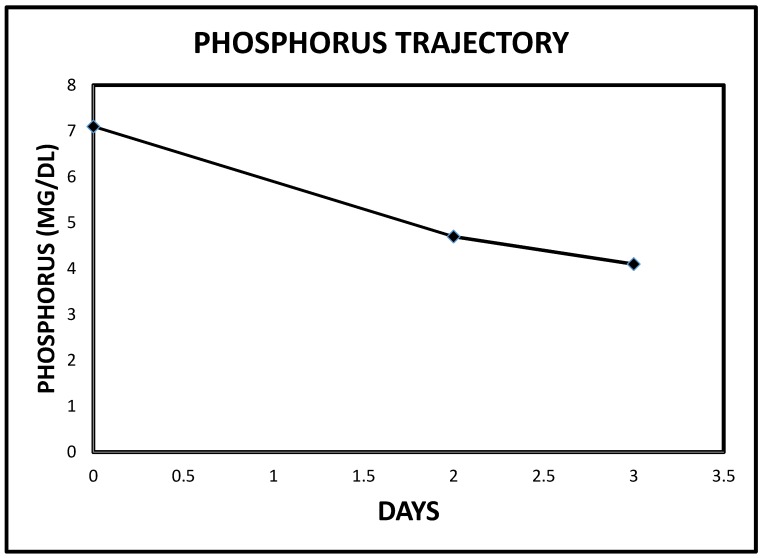
Normalized serum phosphorus from previously hyperphosphatemic levels following intravenous decongestive diuresis in Case 3.

**Figure 14:**
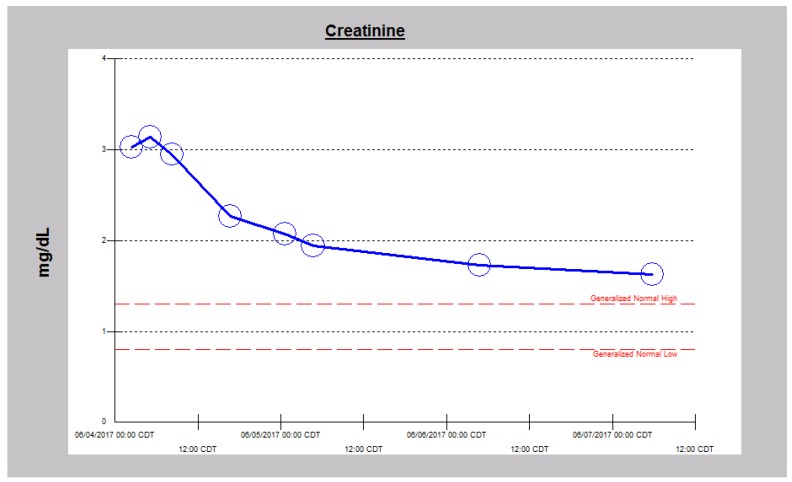
Falling serum creatinine in Case 3 following intravenous decongestive diuresis.

Overnight, the patient exhibited a very prompt, excellent and sustained diuresis with the combination diuretic regimen as he made nearly 10 L of urine the first admission day (Figure 10). He felt much better, was off the BiPaP during the day, was less dyspneic and leg swelling was significantly reduced bilaterally. His weight quickly dropped, and the serum potassium was normalized about 15 h into the admission and continued to decrease thereafter (Figure 11 and Figure 12). Simultaneously, serum bicarbonate had risen sharply, a reflection of contraction alkalosis. Moreover, hyperphosphatemia improved concurrently with acutely falling serum creatinine values (Figure 13 and Figure 14). 

**Figure 15:**
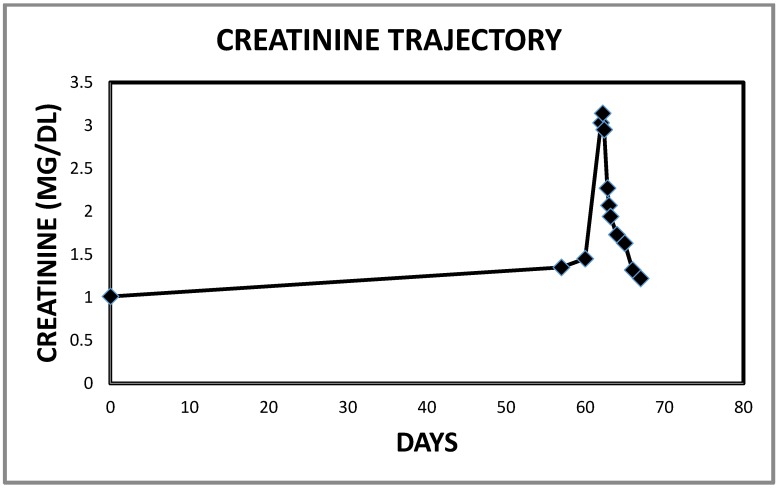
Serum creatinine trajectory in Case 3 leading to and following the use of intravenous decongestive diuresis.

By day 3, he was feeling so much better that intravenous diuretics were discontinued and he was switched to oral Furosemide 40 mg 2 times a day together with oral Metolazone 2.5 mg daily with continued monitoring of intake/output and daily chemistry. The Foley catheter was also discontinued. He continued to improve, lost more weight with loss of anasarca and edema fluids and was discharged after 5 days on the combination oral Furosemide 40 mg twice daily (double the preadmission dose) and oral Metolazone 2.5 mg daily (a new additional diuretic). At discharge, four days later, serum creatinine had fallen to 1.32 mg/dL, phosphorus was normal and he needed potassium supplements for hypokalemia. Besides, the serum bicarbonate was beginning to trend towards normal levels again (Figure 15). Discharge medications included Prednisone taper and he was to complete five days of oral Doxycycline. Lisinopril and Spironolactone remained discontinued at the time of the discharge. He was seen the following week at follow up, in the Nephrology office, on 12 June 2017, much improved, and his weight had come down from a peak of 327 lb to 293 lb, serum creatinine was down to 1.05 mg/dL and he was requiring more potassium chloride supplements, on the same dose of Furosemide (40 mg twice daily) and Metolazone (2.5 mg daily), together with Spironolactone that was reintroduced by his Internist sometime after discharge, at a lower dose of 12.5 mg daily.

## 3. Conclusions

We have demonstrated very impressive degrees of diuresis in three consecutive patients with symptomatic heart failure, associated worsening renal failure consistent with cardiorenal syndrome, following the utilization of the combination of continuous intravenous Furosemide infusion with intravenous Chlorothiazide given every 8–12 h. Symptomatic heart failure relief with loss of edema and anasarca fluid, weight loss, improvement in dyspnea and orthopnea, as well as simultaneous improvement in renal function was achieved in all three patients in our series. This was true even in patient 3, where we demonstrated excellent diuretic response, achieving nearly 10 L of urine in the first twenty-four hours, even in the face of acute kidney injury secondary to contrast nephropathy. This degree of “renal ultrafiltration” far exceeded the mechanical ultrafiltration targets reported in the cardiorenal literature using mechanical dialysis machines [9]; this represents further evidence to attest to the potency of the potential synergy of the simultaneous, concurrent and sequential administration of these two classes of diuretics [10]. 

In a pooled analysis of 208 patients from the DOSE-AHF, ROSE-AHF, and CARRESS-HF trials, the main finding was that intensifying medical therapies with parenteral diuretics while targeting a 3–5 L/d urine output utilizing intravenous Furosemide with oral Metolazone was associated with a greater amount of weight loss and a higher net fluid loss than standard diuretic therapy with intravenous Furosemide alone without any further worsening of renal function. The group that received intensified diuretic therapy had more weight change (−3.4 ± 5.2 lb) and more net fluid loss (1.705 ± 1.417 L) after 24 h than the standard diuretic therapy group (−0.8 ± 3.4 lb and 0.892 ± 1.395 L, respectively; *P* < 0.001 for both) with a slight improvement in renal function (creatinine change −0.1 ± 0.3 vs. 0.0 ± 0.3 mg/dL, respectively; *P* = 0.03) [11]. The addition of thiazide-type diuretics should be considered when a progressive decrease in loop diuretic efficacy is observed with prolonged use (i.e., the braking phenomenon). Furthermore, thiazide-type diuretics are a useful addition in patients with low GFR to maximally boost fractional sodium excretion when nephron perfusion is poor [10,12].

**Figure 16:**
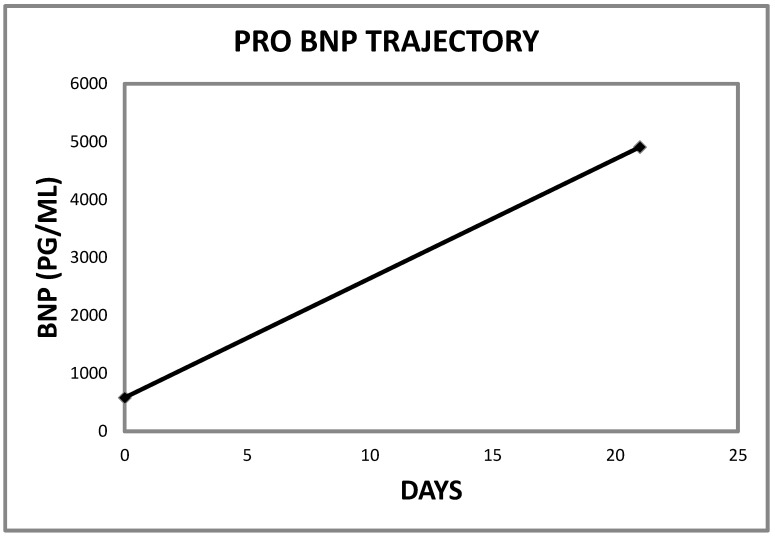
Pro B Nat Pep trajectory in Case 1 immediately preceding the index admission (Reference range for Pro B Nat Pep ≤ 450 pg/mL).

**Figure 17:**
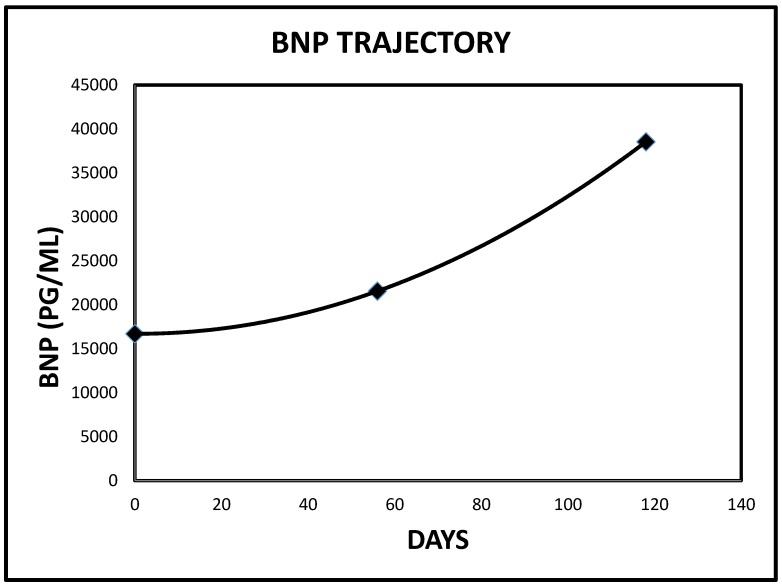
Pro B Nat Pep trajectory in Case 2 immediately preceding the index admission (Reference range for Pro B Nat Pep ≤ 450 pg/mL).

**Figure 18:**
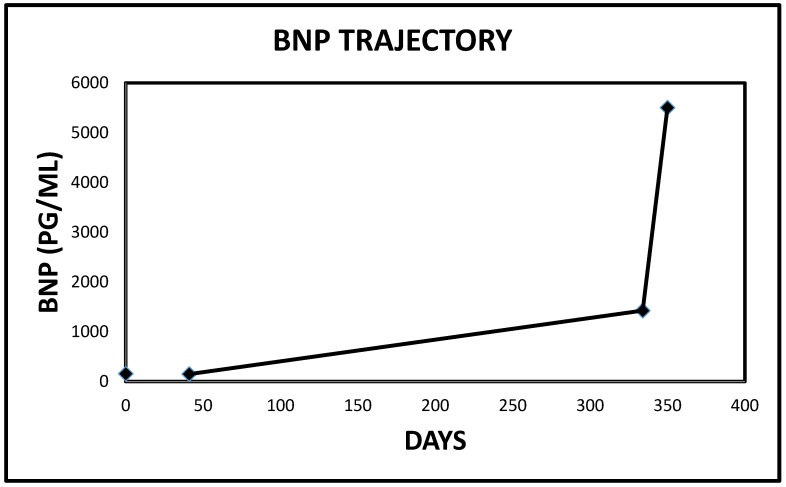
Pro B Nat Pep trajectory in Case 3 immediately preceding the index admission (Reference range for Pro B Nat Pep ≤ 125 pg/mL).

**Figure 19:**
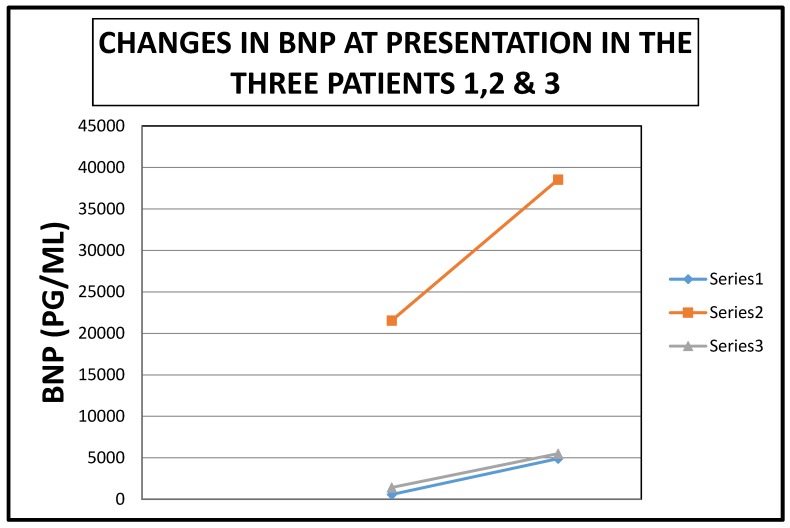
Composite figure showing the acutely rising BNP levels in the three patients at presentation.

Finally, we would wish to emphasize our thrilling observation that all three patients had demonstrated recently rising Pro B Nat Peptide levels prior to each presentation (Table 1, Figure 16, Figure 17, Figure 18 and Figure 19). As a result of these observations, we have proposed a hypothesis that rapidly rising Pro B Nat Peptide levels in patients presenting a priori with cardiorenal syndrome should be seen as a sign of rapidly increasing right atrial pressure and as a result, the subsequent worsening congestive renal failure secondary to renal venous hypertension and therefore is a predictor of positive response to aggressive decongestive diuresis. Moreover, the often more than logarithmic escalations of Pro B Naturetic Peptide level that were observed within short periods of time prior to presentation in our three patients, we posit, is further confirmation of the very heightened central venous pressures operational in these patients on admission that would have clearly translated to exaggerated renal venous hypertension leading to “congestive renal failure” as designated by Ross in 2012 [1]. Of note, among hemodialysis patients, high Pro B Nat Peptide levels have been associated with mortality and generally is known to trend with volume overload [13,14,15,16]. The association of accelerated Pro B Nat Peptide levels with renal and/or mortality outcomes in cardiorenal syndrome not on hemodialysis are unknown. Specific targeted trials are warranted.

Furthermore, the observation of worsening pulmonary hypertension as demonstrated in patient 3 is further testament of the validity of renal venous hypertension in the precipitation and potentiation of renal failure with cardiorenal failure and could also serve as another measurable diagnostic index in this paradigm of care. We would acknowledge that pulmonary hypertension was not evident in patient 1 although she exhibited bi-atrial enlargement. Patient 3 did not have an echocardiogram during the index admission, whereas patient 2 exhibited severe right atrial enlargement.

### 3.1. The Natriuretric Peptides, Heart Failure, Renal Dysfunction and Cardiovascular Disease

The three known natriuretic peptides are atrial natriuretic peptide (ANP), brain natriuretic peptide (BNP), and C-type natriuretic peptide (CNP) and they contribute to the regulation of cardiovascular homeostasis through diuretic, natriuretic, and vasodilatory properties. Among them, ANP has received particular attention because of its effects on blood pressure regulation and cardiac function. Recently, ANP has also received attention as being a possible cardiovascular risk factor, particularly in the context of hypertension, stroke, obesity, and metabolic syndrome [17]. Furthermore, several reports have correlated elevated BNP biomarker levels with diuretic resistance, hypotension, hyponatremia, longer length of stay, greater inotrope use, and substantially worse survival in patients with renal dysfunction and heart failure, consistent with cardio-renal syndrome [18]. More recently experimental evidence using BNP-deletion has lent stronger impetus to the role of BNP (its absence) in the pathogenesis of hypertension [19]. Finally, the question of renal hyporesponsiveness to high BNP levels and cardiorenal outcomes has been investigated. Egom et al. have observed that several mechanisms have been proposed to explain renal hyporesponsiveness in heart failure, including, but not limited to, decreased renal BNP availability, down-regulation of natriuretic peptide receptors, and altered BNP intracellular signal transduction pathways. Thus, a better understanding of renal hyporesponsiveness in failure is required to devise strategies to develop novel agents and technologies that directly restore renal BNP efficiency. It is hoped that development of these new therapeutic approaches will serve to limit sodium retention in patients with heart failure, which may ultimately delay the progression to overt heart failure [20]. However, these topics, albeit exciting and intriguing, remain beyond the scope of our current manuscript.

### 3.2. Diuretic Resistance in Cardiorenal Syndrome

Verbrugge et al. in a 2016 review had acknowledged that diuretic resistance in acute heart failure has emerged as a powerful predictor of adverse outcome, which is often independent of underlying glomerular filtration rate [12]. One of the conclusions was that the addition of thiazide-type diuretics should be considered when a progressive decrease in loop diuretic efficacy is observed with prolonged use (i.e., the braking phenomenon). We posit that our three case presentations support the clinical reality of this braking phenomenon [10]. Moreover, according to Verbrugge et al., in a 2015 prospective, single-center cohort study, consecutive patients with decompensated heart failure (*n* = 54) and left ventricular ejection fraction 45% received protocol-driven diuretic therapy until complete disappearance of congestion signs. Urine was collected during three consecutive 24-h intervals. Natriuretic response was defined as absolute natriuresis (mmol) per mg of intravenous bumetanide administered [12]. Natriuresis was 146 mmol (76–206 mmol), 74 mmol (37–167 mmol) and 74 mmol (53–134 mmol) per mg intravenous bumetanide administered during the first, second and third 24-h interval, respectively [12]. Diastolic blood pressure (beta = 23.048 ± 10.788; *p*-value = 0.036), plasma aldosterone (beta = −25.722 ± 11.560; *p*-value = 0.029), and combination therapy with acetazolamide (beta = 103.241 ± 40.962; *p*-value = 0.014) were independent predictors of the natriuretic response [12]. Patients with a stronger natriuretic response demonstrated more pronounced decreases in plasma NT-proBNP levels (*p*-value = 0.025) [12]. This would support our hypothesis that acutely rising BNP levels at presentation with diuretic resistance may predict better decongestive therapy responses. More studies are warranted.

Finally, it is our submission that in selected patients presenting with acute decompensating heart failure concurrent with worsening renal failure and other related electrolyte and metabolic derangements, the judicious use of simultaneously administered continuous Furosemide infusion and intravenous Chlorothiazide could result in very significant cardiorenal salvage. It would appear that escalating levels of Pro B Naturetic Peptide may represent a good prognosticator for a good response to this form of decongestive diuresis. There is evidence in the literature that cardiorenal patients with a stronger natriuretic response demonstrated more pronounced decreases in plasma NT-proBNP levels (*p*-value = 0.025) [12]. This is yet another set of evidence supporting the significant role of congestive renal failure in our understanding and proper management of patients with cardiorenal syndrome [1,2,3,4,5,6,7,21]. Such therapeutic maneuvers would prove invaluable to practitioners in resource-poor settings without access to mechanical ultrafiltration with dialysis or similar equipment.

## Figures and Tables

**Table 1:** Changes in BNP levels in the three patients at presentation versus the penultimate BNP value.

Patient	BNP Level at Presentation	Penultimate BNP Level	Reference Range	Interval between BNP Tests (Days)
1	4907	581	<450 PG/ML	21
2	38,547	21,557	<450 PG/ML	63
3	5503	1426	<125 PG/ML	16
